# Fatigue Reliability Characterisation of Effective Strain Damage Model Using Extreme Value Distribution for Road Load Conditions

**DOI:** 10.3390/ma16010456

**Published:** 2023-01-03

**Authors:** Lennie Abdullah, Salvinder Singh Karam Singh, Shahrum Abdullah, Ahmad Kamal Ariffin, Syifa Syuhaidah Meor Zainal

**Affiliations:** Department of Mechanical and Manufacturing Engineering, Faculty of Engineering and Built Environment, Universiti Kebangsaan Malaysia, Bangi 43600, Selangor, Malaysia

**Keywords:** fatigue life, fatigue reliability, fracture, strain load, cycle sequence effect

## Abstract

The aim of this paper is to characterise the fatigue reliability for various random strain loads under extreme value distribution while considering the cycle sequence effect condition in fatigue life prediction. The established strain–life models, i.e., Morrow and Smith–Watson–Topper, considered a mean stress effect and strain amplitude; nevertheless, it excluded the load sequence effect, which involves the fatigue crack closure that is subjected to overload or underload. A FESEM-EDX analysis is conducted to characterise the failure features that occurred on the leaf spring. A finite element is simulated to determine the critical region in order to obtain the strain load behaviour. In addition, the strain signal is captured experimentally at 500 Hz for 100 s under operating conditions for three different road loads based on the critical location obtained from the finite element analysis. The fatigue life correlation shows that the Pearson correlation coefficients are greater than 0.9, which indicates the effective strain damage model is linearly correlated with the strain–life models. The fatigue life data are modelled using extreme value distribution by considering the random strain loads as extreme data. The reliability rate for the fatigue life is reported to be more than 0.59 within the hazard rate range of 9.6 × 10^−8^ to 1.2 × 10^−7^ based on the mean cycle to the failure point. Hence, the effective strain damage model is proposed for a fatigue reliability assessment under extreme conditions with higher reliability and provides fatigue life prediction when subjected to cycle sequence effects.

## 1. Introduction

Automotive components or structures are typically subjected to random loads during operating conditions due to various road profiles. A dynamic stress or strain reaction generated by a random load on suspension during actual driving conditions can be detected on the basis of time and frequency domains [1]. Fatigue failure due to repetitive loading starts with crack dissemination until the ensuing fracture. This generally occurs in automotive components such as coil springs, anti-roll bars and the crankshaft [2,3,4]. Fatigue failure in automotive suspension systems has been characterised using chemical composition analysis, hardness testing and macroscopic investigation on coil springs [5], shackle brackets [6], steering knuckles [7] and leaf springs [8] by subjecting them to static loading [9] and fatigue tests [10]. A leaf spring is a slender arc-shaped component with a long and narrow rectangular cross-section steel plate, and is the commonly used suspension system for light and heavy commercial vehicles. Plain carbon steel (0.9–1.0% carbon) is the typical material of leaf springs that requires heat treatment after the forming process and provides high strength, high displacement deformation and enhanced fatigue properties [11]. Leaf springs are connected from the vehicle frame to the axle through a shackle on one end and a moveable pin joint on the other, absorbing vertical loads and impacts exerted from various road conditions. Vibration absorption is stored as strain energy and released to ensure secure, safe and comfortable driving [12]. Vertical loads will spread along the length of the leaf spring and produce constant forces, resulting in comfort when vibrations are absorbed. Leaf springs are exposed to cyclic fatigue loading because of irregular road surfaces. Thus, this component needs to support more than 10^7^ loading cycles under a minimal weight within its in-service life [13].

The probabilistic approach for automotive components’ fatigue failure under random road loads results is identified as non-Gaussian with non-stationary distributions. Manouchehrynia et al. [14] demonstrated the strain–life probabilistic modelling in the fatigue reliability evaluation of a coil spring using a goodness-of-fit test and Gumbel distribution as optimal for random loads. The probability of failure related to automotive components must be correctly determined when predicting the service life on the basis of fatigue loading [15]. A durability analysis is essential to ensure that structures or components are safe under random amplitude loads and have a long service life. This evaluation is implemented according to the fatigue damage theory that involves a damage accumulation rule and an appropriate cycle counting technique [16]. The majority of studies on leaf springs focused on finite element analysis (FEA) for material substitution purposes [17,18], reliability-based fatigue design [12,13] and fatigue life prediction [19]. Leaf springs are also a type of suspension component that requires a fatigue analysis with random loads under varying road profiles [19]. A conventional fatigue reliability assessment of a structure typically involves numerical or test analysis methods or a combination of both.

A fatigue reliability test on a structure is proposed to evaluate the probability of structural damage based on design parameters as the variable input. Several studies on probabilistic reliability evaluation have been conducted. Guillal et al. [20] used several distributions, such as normal, lognormal, and Gumbel and Frechet, on pipelines to detect corrosion and crack defects. Anderson and Daniewicz [21] demonstrated that Gumbel distribution parameters influence the characteristics of fatigue life of 7075-T651 aluminium alloys. Le et al. [22] investigated the fatigue behaviour of the Ti-6Al-4V alloy by examining the influence of defects on the size effect using the Gumbel distribution and discovered that the pore size of crack initiation is associated with the Gumbel distribution. Bag et al. [23] used the Gumbel distribution to describe inclusion size at the fatigue crack initiation in shot-peening 300M steel and showed that the predicted maximum inclusion size is appropriately represented by the Gumbel distribution. Szmytka et al. [24] utilised the Gumbel distribution to identify the nodular size in determining low cycle fatigue life span and successfully classified the maximum nodule size under the Gumbel distribution.

The Gumbel distribution is suitable for random excessive in predicting fatigue life [23], mechanical failure [25] and corrosion of structure [20], as well as the application of environmental data [26]. Kepra and Kepra [8] probabilistically calculated fatigue life based on the welded joint failure of a trolleybus rear axle by describing the probability of fatigue failure and reliability level. Fu et al. [27] characterised fatigue reliability under variable loads by clustering the probability density evolution model of a wind turbine structure. Hu et al. [28] predicted a fatigue life thermal-based model of ball grid arrays by considering a load sequence effect that focuses on fatigue crack growth in electronic devices and enhancing a fatigue life prediction model. Fatigue life prediction based on fracture mechanics under random loads, such as measuring crack growth rate, is dependent on specific load history due to the difficulty of cycle sequence effects [29]. Maierhofer et al. [30] revealed that short crack behaviour and the effect of load sequence associated with random loads are factors that primarily contribute to fatigue crack growth on railway axles.

Studies on characterising fatigue reliability using the Gumbel distribution (extreme value distribution) for leaf springs under data random load strain that consider the cycle sequence effect are still inadequate. This is because prior studies mainly assessed failure using the Weibull distribution, which does not provide the fatigue reliability characteristic under extreme distribution values [31,32,33]. The work involved a load sequence effect in fatigue life calculation that has been performed by Chin [34], with this model well fitted with established strain–life models by plotting the Weibull probability plot. A Gumbel distribution on an automotive component is applied because the related study predicting the fatigue life span needs an enormous data case in order to obtain an accurate result [35]. The appropriate frequency rate depends on the measured signal to ensure that sufficient information is captured. This study aims to assess the fatigue life data obtained from strain–life models of random strain data from several road conditions. Fatigue life is determined under various strain–life models to obtain accurate results in a fatigue reliability assessment. Thus, the effective strain damage (ESD) model is proposed to probabilistically characterise fatigue reliability using extreme value distribution on random road loads for risk assessment.

## 2. Materials and Methods

**Figure 1:**
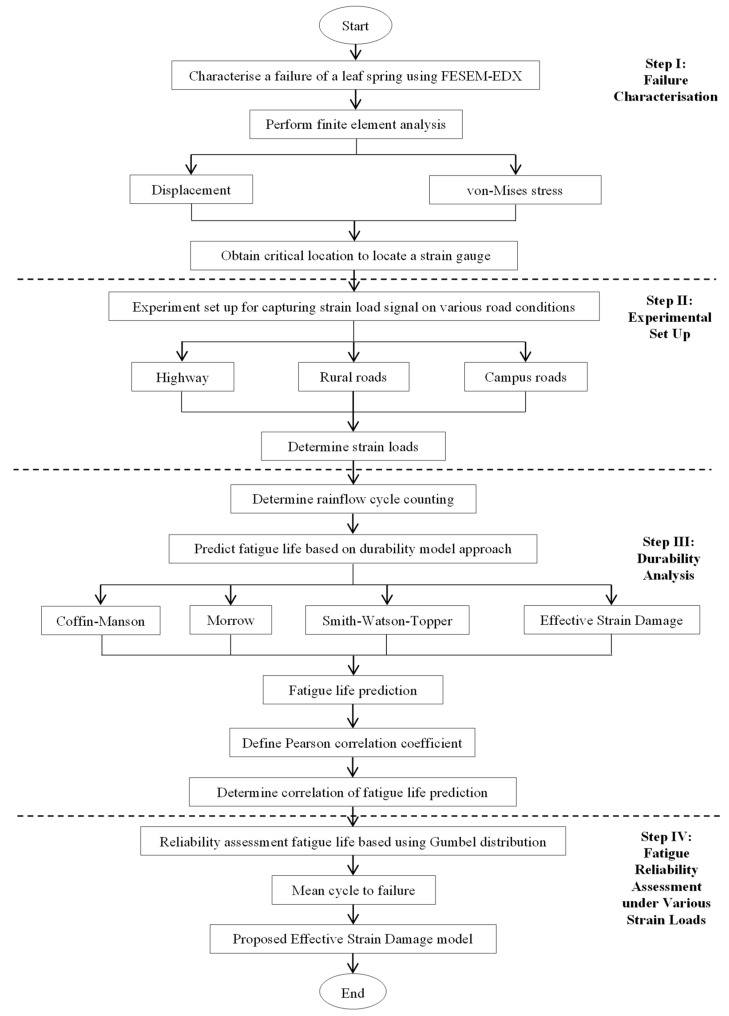
Process flow for fatigue-based reliability under random road load conditions.

The methodological framework for the reliability assessment associated with the fatigue strain–life model-generated strain loads shown in Figure 1 is divided into the following steps:

Step 1: Failure characterisation: failure crack investigation and FEA
Step 2: Experimental setup: data acquisition and strain data characterisation
Step 3: Durability analysis: fatigue life prediction and correlations
Step 4: Fatigue reliability: fatigue strain–life reliability assessment

### 2.1. Step I: Failure Characterisation Using FESEM-EDX

**Figure 2:**
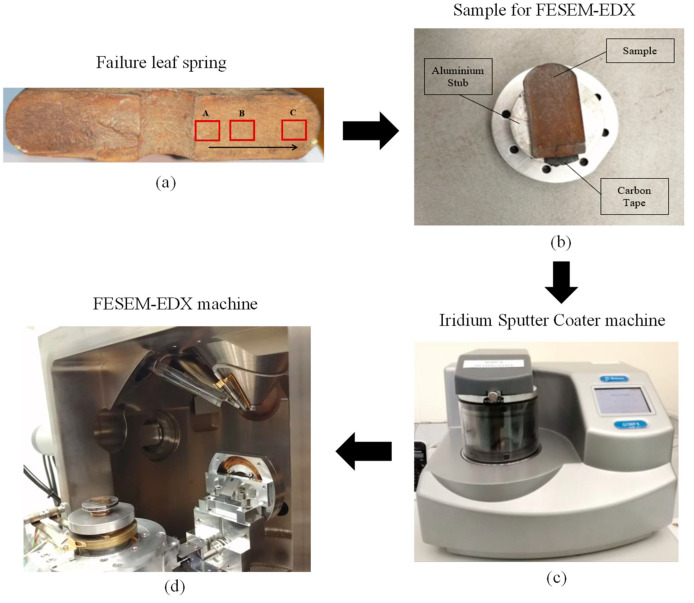
Fracture identification in FESEM-EDX test for (**a**) Failure leaf spring; (**b**) Sample for FESEM-EDX; (**c**) Iridium Sputter Coater machine; (**d**) FESEM-EDX machine.

The damaged component may experience a short service life due to high mass, low damping and being easily oxidised or rusted [36,37]. A damaged leaf spring of a bus is used as a case study to identify its fracture mode. Field emission scanning electron microscopy associated with energy dispersive X-ray spectroscopy (FESEM-EDX) is performed to identify the failure condition and material content of a component. A sample is cut at the fractured area (Figure 2a) and then placed in an oven for drying before any test is performed. Figure 2b shows the FESEM-EDX sample.

The sample is then coated with a layer of iridium (Ir) in a vacuum chamber with inert argon gas added into the chamber through a fine control valve (leakage) using an iridium sputter coater machine, as illustrated in Figure 2c. The conductive coating material is important in the FESEM-EDX test because it allows the necessary removal of recognition electrons to produce an acceptable image during the test. The sample was then placed in a FESEM-EDX machine after iridium coating, as shown in Figure 2d.

#### Finite Element Modelling for Assessing Critical Region

**Figure 3:**
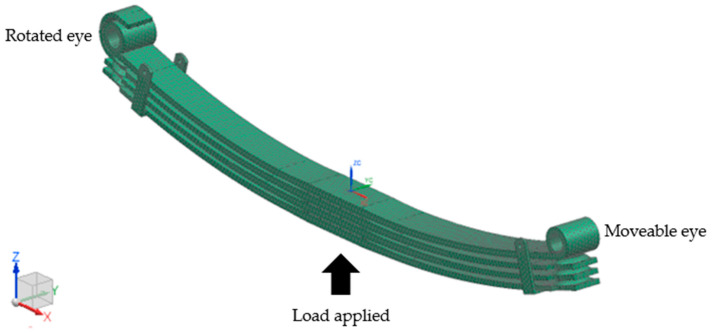
FE model of a leaf spring.

FEA was performed on the leaf spring geometry to identify the critical location of the strain gauge. Three-dimensional (3D) isoparametric tetrahedron elements attained 51,377 nodes and 100,725 elements in a 10 mm mesh on the leaf spring geometry model, and mesh convergence was conducted to obtain reliable results using SimcenterTM 3D software (Figure 3). For boundary conditions, one eye of the leaf spring was secured to act as a rigid shackle with a rotating movement, as shown in Figure 3. Meanwhile, the other eye was set as a moveable shackle in the *x*-axis direction. The load was applied at the middle of the leaf spring in an upward vertical movement. The total mass weight of a bus, including a full load of passengers, is 14,200 kg. A quarter of the total mass weight is used as the consideration to calculate the deformation and von Mises stress. Thus, the applied load is set to 34.815 kN per leaf spring for static analysis purposes. SAE5160 carbon steel, a commonly used material for leaf springs, was used as the boundary condition. The mechanical properties of the applied SAE5160 carbon steel are as follows [38]: ultimate tensile strength (*S_u_*) of 1584 MPa, yield strength (*S_y_*) of 1487 MPa, modulus of elasticity (*E*) of 207 GPa, density at a range of 7.7–8.03 × 10^3^ kg/m^3^ and Poisson’s ratio of 0.27. Cyclic properties of the applied SAE5160 carbon steel are as follows: fatigue strength coefficient (*σ’_f_*) of 2063 MPa, fatigue strength exponent (*b*) of −0.08, fatigue ductility exponent (*c*) of −1.05, fatigue ductility coefficient (*ɛ’_f_*) of 9.56 MPa, cyclic strain hardening exponent of 0.05 and cyclic strength coefficient of 1940 MPa. Figure 3 illustrates the FE model of the leaf spring.

### 2.2. Step II: Experimental Setup for Strain Load Exertion on Various Road Load Conditions

**Figure 4:**
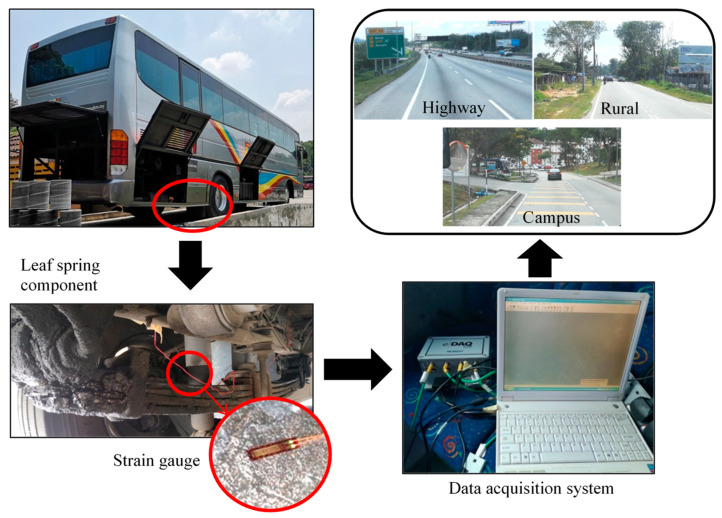
Experimental setup for strain load data collection for various road conditions.

A commercial bus was driven on a smooth highway, a slightly potholed rural road and a bumpy campus road of Universiti Kebangsaan Malaysia to collect various road loads at speed ranges of 70–80, 50–60 and 30–40 km/h, respectively. The speed adheres to the National Speed Limit Order 1989 of Malaysian roads, while road tests conform to the ISO8608 standard for road surfaces of a rough level [39]. Data collection was repeated three times to ensure the preciseness of the data extracted. A strain gauge was installed on top of the leaf spring of the bus with a strong adhesive to gather strain data, as illustrated in Figure 4. The strain gauge was subsequently attached to a data logger and a laptop for real-time data monitoring. A sampling rate of 500 Hz was chosen for data extraction to store all the necessary information that was acquired [40]. Haiba et al. [41] stated that a frequency of 500 Hz is sufficient in determining the damage to automotive components. Figure 4 displays the experimental setup of the attached strain gauge on the leaf spring component and connection to the data acquisition system.

### 2.3. Step III: Durability Analysis

A durability assessment is used to analyse important issues and ensure that mechanical structures and components subjected to various loads in long usage periods are safe [42]. This analysis gathers information on the fatigue properties of the material, the geometry of the component and the operating load. Fatigue life prediction involves a loading history, damage cycle count and estimation of the summation damage [43]. The physics-based model, such as cyclic softening of P91 steel under low cycle fatigue at a higher temperature, has been proposed by Egner et al. [44] and considers two approaches in fatigue damage modelling by comparing strain-controlled ductile damage identification and entropy-based methods. These models are based on thermomechanical fatigue behaviour due to the effects of temperatures change, but are lacking in describing a specific procedure [45].

Elsewhere, the proposed conventional strain–life model mainly focuses on the effects of mean stresses by which it has the capability of predicting fatigue life with good accuracy [46,47]. The strain–life approach is chosen in this study because the leaf spring will start to fail when a crack initiates and a small crack growth occurs [48]. The Coffin–Manson model describes the relationship between plastic and plastic components as follows [48]:

(1) $$\varepsilon_{a}=\frac{\Delta \varepsilon }{2}=\frac{{\sigma^{\prime }}_{f}}{E}{\left(2N_{f}\right)}^{b}+{\varepsilon^{\prime }}_{f}{\left(2N_{f}\right)}^{c},$$

where Δ *ɛ*/2 is the total strain amplitude (*ɛ_a_*), *σ*′*_f_* is the fatigue strength coefficient, *ε*′*_f_* is the fatigue ductility coefficient, *E* is Young’s modulus of elasticity, *N_f_* is the fatigue life, *b* is the fatigue strength exponent and *c* is the fatigue ductility exponent.

The mean stress values of the mechanical structure commonly represent the fatigue behaviour according to in-service cyclic loads [49]. Morrow’s rule specifies that the mean stress is primarily affected in the initial stage of loading or for high fatigue life [50]. The strain amplitude of elastic in different types of fatigue life impacts the total life. Morrow quantifies the link between the mean stress level and fatigue life as follows [50]:

(2) $$\varepsilon_{a}=\frac{\Delta \varepsilon }{2}=\frac{\left({\sigma^{\prime }}_{f}-\sigma_{m}\right)}{E}{\left(2N_{f}\right)}^{b}+{\varepsilon^{\prime }}_{f}{\left(2N_{f}\right)}^{c},$$

where *ɛ_a_* is the total strain amplitude and *σ_m_* is the mean stress. The Morrow model estimates the influence of the mean stress on the extent of the lifespan, in which elastic strain amplitudes are dominant.

The mean stress effect based on Smith–Watson–Topper (SWT) is dominated by the product of maximal tensile stress and strain amplitude [51]. The SWT model assumes that the *ɛ_a_σ_max_* parameter at a particular life is constant for different combinations of strain amplitudes and maximum stresses. The SWT relationship is expressed as follows [51]:

(3) $$\frac{\Delta \varepsilon }{2}\sigma_{\max}=\frac{{\left({\sigma^{\prime }}_{f}\right)}^{2}}{E}{\left(2N_{f}\right)}^{2b}+{\sigma^{\prime }}_{f}{\varepsilon^{\prime }}_{f}{\left(2N_{f}\right)}^{b+c}$$

Some approaches provide enhancement but can be difficult to apply to commonly used fatigue life prediction programmes. Hence, ESD, an extensive model of strain–life fatigue damage, is presented in this study [52]. This technique for obtaining the source of crack growth and closing has been successfully used with several materials, geometries of components, load spectra, magnitudes of strain and mean-strain effects. The proposed model is expressed as follows [52]:

(4) $$E\Delta \varepsilon^{*}=A{\left(N_{f}\right)}^{B},$$

where Δɛ* is the range of net effective strain for a closed hysteresis loop linked to fatigue crack growth, *A* and *B* are material constants and *N_f_* is the number of cycles before failure. The magnitude of *E*Δɛ* for a specific cycle is the crack-opening stress function level (*S_op_*) that indicates prior stress and strain magnitudes in the loading history. *E*Δɛ* can be improved as follows [52]:

(5) $$E\Delta \varepsilon^{*}=E\left(\varepsilon_{max}-\varepsilon_{op}\right)-E\varepsilon_{i},$$

where *ε_max_* and *ε_op_* are the maximal and crack-opening strains of a specific cycle, respectively, and *ε_i_* is the intrinsic fatigue limit strain range based on variable loads. The decay parameter (*m*) is applied to ascertain the alteration in the crack-opening stress in two adjacent cycles by considering the impacts of cycle sequence in the fatigue life calculation. Δ*S_op_* is expressed as follows [52]:

(6) $$\Delta S_{op}=m\left(S_{ss}-S_{cu}\right),$$

where *S_cu_* is the current opening stress and *S_ss_* is the steady-state opening stress. *S_cu_* is shown as the *S_op_* value of the previous cycle. *S_ss_* is expressed as follows [52]:

(7) $$\Delta S_{ss}=\alpha S_{max}\left(1-\left(\frac{S_{max}}{S_{y}}\right)\right)+\beta S_{min},$$

where *α* and *β* are material constants, *S_max_* is the maximal stress of the previous largest cycle in the time history, *S_min_* is the minimal stress of the prior largest cycle and *S_y_* is the cyclic yield stress [52].

Fatigue life (*N_i_*) for each cycle is expressed as follows [52]:

(8) $$N_{i}={\left(E\Delta \varepsilon^{*}/A\right)}^{1/B}$$

The linear damage rule of the Palmgren–Miner (PM) method was applied for random loading [53]. A particular block is the sum of cycles on every block *N_c_* and *D* is the damage, which can be expressed as follows [54]:

(9) $$D=\sum \;\frac{n_{i}}{N_{i}},$$

where *n_i_* is the number of cycles used at level *i* on the stress amplitude and *N_i_* is the sum of cycles [54].

### 2.4. Step IV: Fatigue Reliability Assessment under Various Strain Loads

The extreme value, or Gumbel, is used on the distribution model of the maximal or minimal number of samples in various distributions [55]. A fixed number of data are compiled in set form and repeated multiple times, whereby the maximal value from each set follows the Gumbel distribution with a cumulative distribution function (CDF) for the number of cycles to failure *N_f_* (*F(N_f_*)) expressed as follows [55]:

(10) $$F\left(N_{f}\right)=e^{-e^{-\left(N_{f}-\lambda \right)/\delta }},$$

where *λ* is the location parameter and *δ* is the scale parameter.

The mean value for the Gumbel distribution is calculated as follows [21]:

Mean = *λ* + *γδ*, (11)

where *γ* is the Euler’s constant equal to 0.5776 [21].

The probability density function (PDF) of the Gumbel distribution (*f*(*N_f_*)) is expressed as follows [55]:

(12) $$f\left(N_{f}\right)=\frac{1}{\delta }e^{\left(\frac{N_{f}-\lambda }{\delta }+e^{-\frac{N_{f}-\lambda }{\delta }}\right)}$$

The curve of the Gumbel distribution is sloped to the left, while the PDF has no shape parameter and remains unchanged. Meanwhile, the location parameter (*λ*) in the PDF is similar to the mode but varies on the basis of the median and mean. The PDF shifts to the left as *λ* decreases and vice versa [55].

The hazard function of the Gumbel distribution *h(N_f_*) is a significant quantity for characterising the life phenomena and is expressed as follows [56]:

(13) $$h\left(N_{f}\right)=\frac{1}{\delta }e^{\left(-\frac{N_{f}-\lambda }{\delta }\right)}$$

The reliability of the Gumbel distribution *R(N_f_*) is expressed as follows [56]:

(14) $$R\left(N_{f}\right)=\frac{1}{\delta }e^{-\left(e^{-\frac{N_{f}-\lambda }{\delta }}\right)}$$

The Gumbel method is the probability technique used to model extreme events, such as random data, while the Gumbel distribution focuses on applications of severe value theory for engineering difficulties [56].

## 3. Results

This section discusses the findings related to the fatigue strain–life model-generated strain loads associated with a reliability assessment. A sample of fracture leaf spring was analysed under field emission scanning electron microscopy. The finite element analysis was performed to locate a critical region on a leaf spring. Random strain data were then collected under various road profiles. The fatigue life was predicted according to strain–life models. Conventional and linear correlations of fatigue life models were conducted by obtaining the coefficient value. Finally, the reliability assessment was conducted associated with the model-based fatigue strain–life.

### 3.1. Failure Characteristics

**Figure 5:**
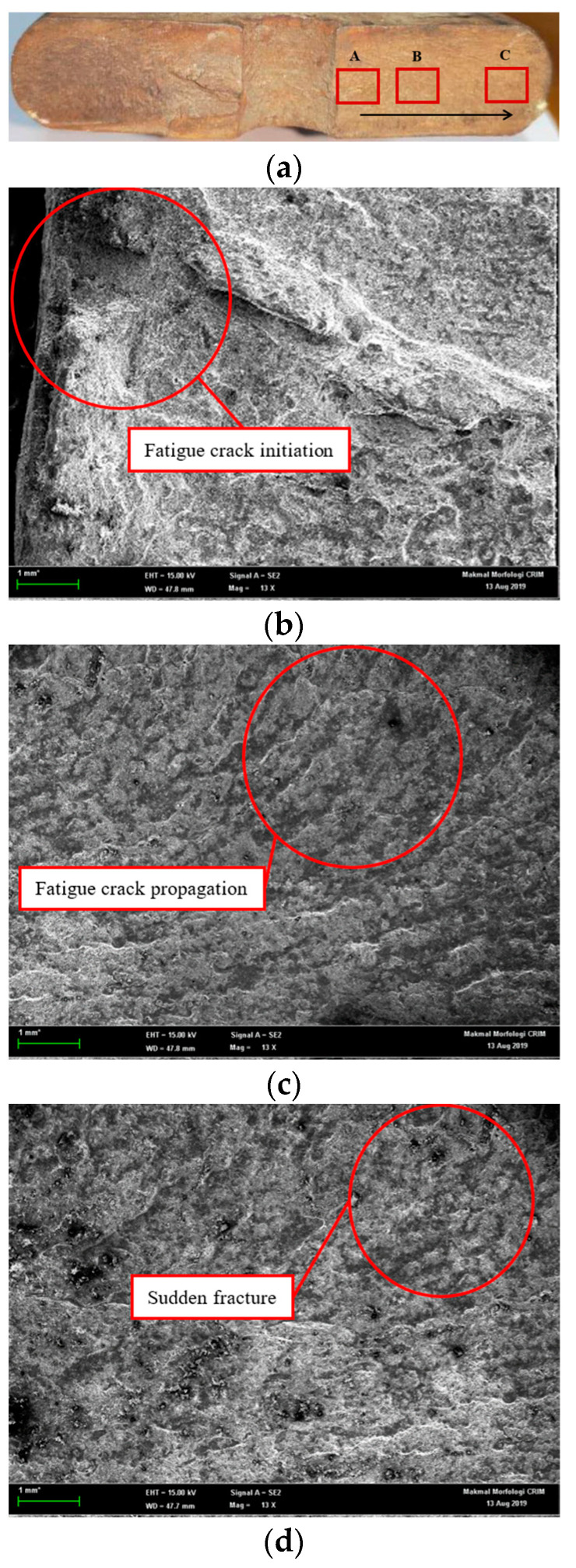
Fractography of the leaf spring: (**a**) Crack region; (**b**) Fatigue crack initiation at region A; (**c**) Fatigue crack propagation at region B; (**d**) Sudden fracture at region C.

The fracture surface of the failed leaf spring was examined, with its chemical composition determined using FESEM-EDX. The damage on the leaf spring was observed with a microscope, starting from the crack occurrence on the surface discontinuity on the U-bolt, as this region is where stresses are concentrated on the leaf spring. Fatigue crack dissemination ensues from region A, spreads to region B and continues to region C as the crack closure of the fracture area, as shown in Figure 5a. Figure 5b illustrates the fatigue crack initiation area on the damaged U-bolt section of the leaf spring.

Figure 5c displays the fatigue crack propagation with beach marks in region B. These marks are caused by a random load applied on the component during the service life of the leaf spring. Sufficiently high cycles can cause yields that will produce a line of beach marks. Figure 5d indicates the failed end of the leaf spring where final stress cycles on the component made it impossible for the leaf spring to bear the load applied, leading to a sudden fracture. The chemical configuration of SAE 5160 materials tested for energy-dispersive X-ray spectroscopy (EDX) is listed in Table 1. It shows that the chemical composition of a sample test has a significant value with a standard SAE 5160, which may be caused by the prolonged time in-service affecting the chemical properties of the material.

#### Finite Element Analysis Leaf Spring under Static Load

**Figure 6:**
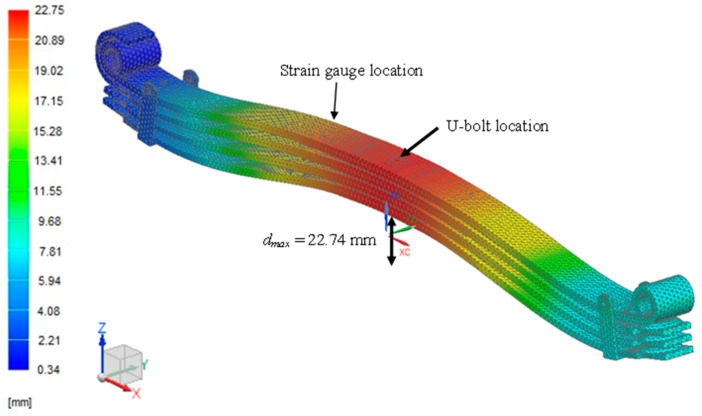
Critical region at maximum load.

**Figure 7:**
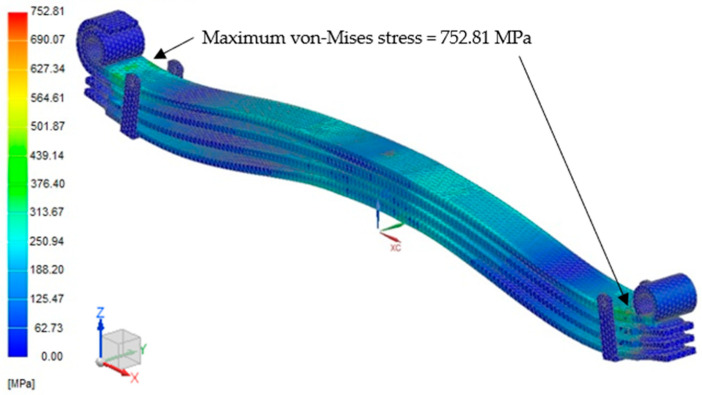
Von Mises stress mapping under maximum load.

FEA was used to obtain the critical hotspot on the leaf spring. A vehicle load of 34.814 kN was applied using the FE model, with the total vehicle mass divided by four to mimic the applied load for each leaf spring. Figure 6 shows the critical hotspot region with a maximum deformation at 22.74 mm. This result is consistent with the FE model in Figure 6, where the critical region is at the U-bolt area, which is located on a red contour, as the effects of shear stresses on the leaf spring subsurface are due to cyclic loading [57]. The maximum critical region based on the von Mises stress shown in Figure 7 is determined as the location where the strain gauge is attached in the U-bolt area with space constraints. The maximum von Mises stress of 752.81 MPa is still lower than the ultimate tensile strength of 1584 MPa. Thus, this analysis can successfully identify the critical region.

### 3.2. Strain Load History Characteristics

**Figure 8:**
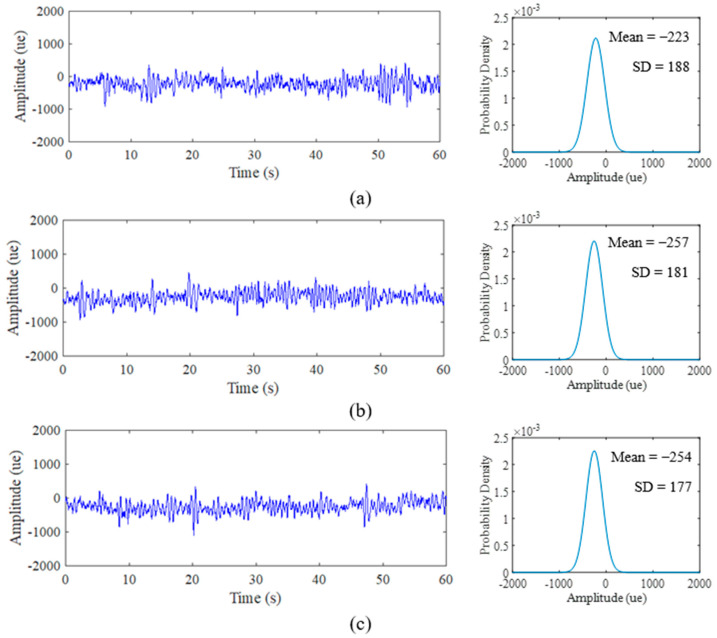
Time history road load obtained from experimental and PDF of: (**a**) Highway Day 1; (**b**) Highway Day 2; (**c**) Highway Day 3.

**Figure 9:**
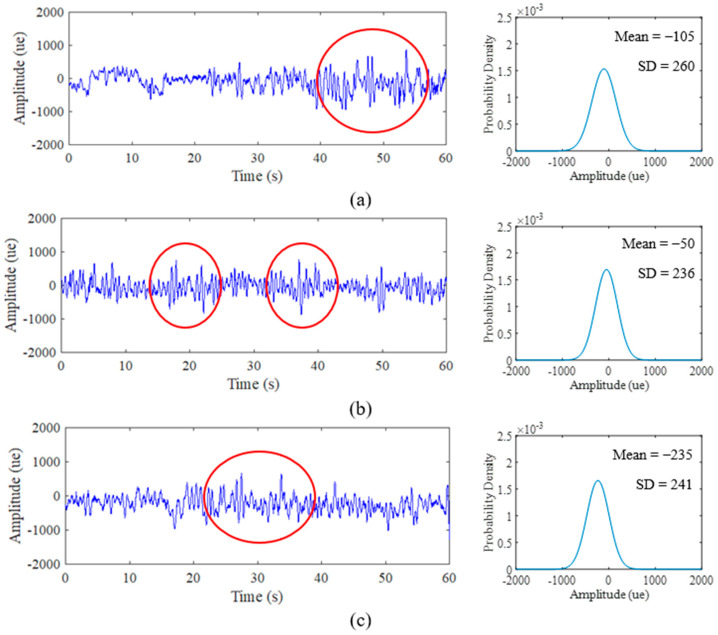
Time history road load obtained from experimental and PDF of: (**a**) Rural Day 1; (**b**) Rural Day 2; (**c**) Rural Day 3.

**Figure 10:**
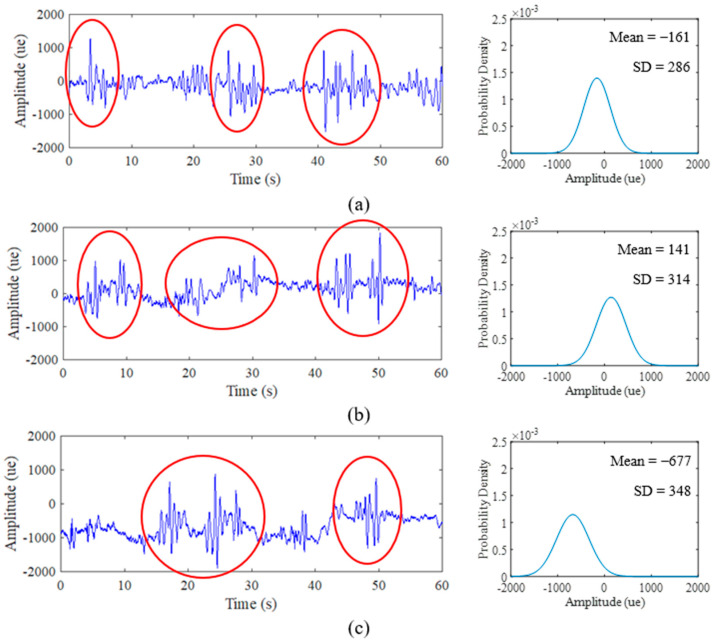
Time history road load obtained from experimental and PDF of: (**a**) Campus Day 1; (**b**) Campus Day 2; (**c**) Campus Day 3.

Various strain data extracted for the duration of 60 s at a 500 Hz sampling rate resulted in 30,000 discrete data points. In order to collect a sufficient amount of information from strain data, 500 Hz is enough to be set for road test purposes [40]. Figure 8, Figure 9 and Figure 10 display the time history and PDF of the highway, rural and campus roads, respectively, with each road type exhibiting three different sets of data collection. The unit of collected data is the microstrain (*µɛ*). The comparison between road types shows that several amplitude peaks of campus data are higher than those of rural and highway data, as highlighted by the red circles in Figure 9 and Figure 10. Campus data also exhibit several high amplitude events compared with rural data due to braking activity at humps found along the campus road.

The PDF also exhibits similar curve shape behaviour in accordance with signal characteristics. The narrow bell-shaped curve of the highway data PDF indicates the consistent low amplitude range that can be interpreted with the SD parameter. The broad bell-shaped curve of the campus data PDF is due to several maximum amplitude ranges that influence the spread of the distribution plot. The behaviour of the PDF plots can be clarified through global statistical parameters. Notably, the behaviour of this strain data is due to the road profile that consistently affects the vehicle with complex circumstances in random vehicle speeds, loads and road characteristics [58]. This attribute can be a factor towards the existence of high-amplitude strains.

Global statistical attributes of the mean, SD and skewness are summarised in Table 2. The mean value indicates the location of the distribution peak on the PDF plot, while the SD value evaluates the extent of spread in the distribution. Meanwhile, the skewed value explains the shift of the distribution towards the left if the value is negative and vice versa. The comparison of PDF plots in Figure 8 and Figure 9 showed that highway data are consistent, rural data are slightly different in locations and campus data vary significantly due to the influence of high amplitude ranges on campus data. The mean value produces negative and positive values that indicate the respective tension and compression movements endured by the leaf spring during its service life.

### 3.3. Life Assessment for Various Road Conditions

**Figure 11:**
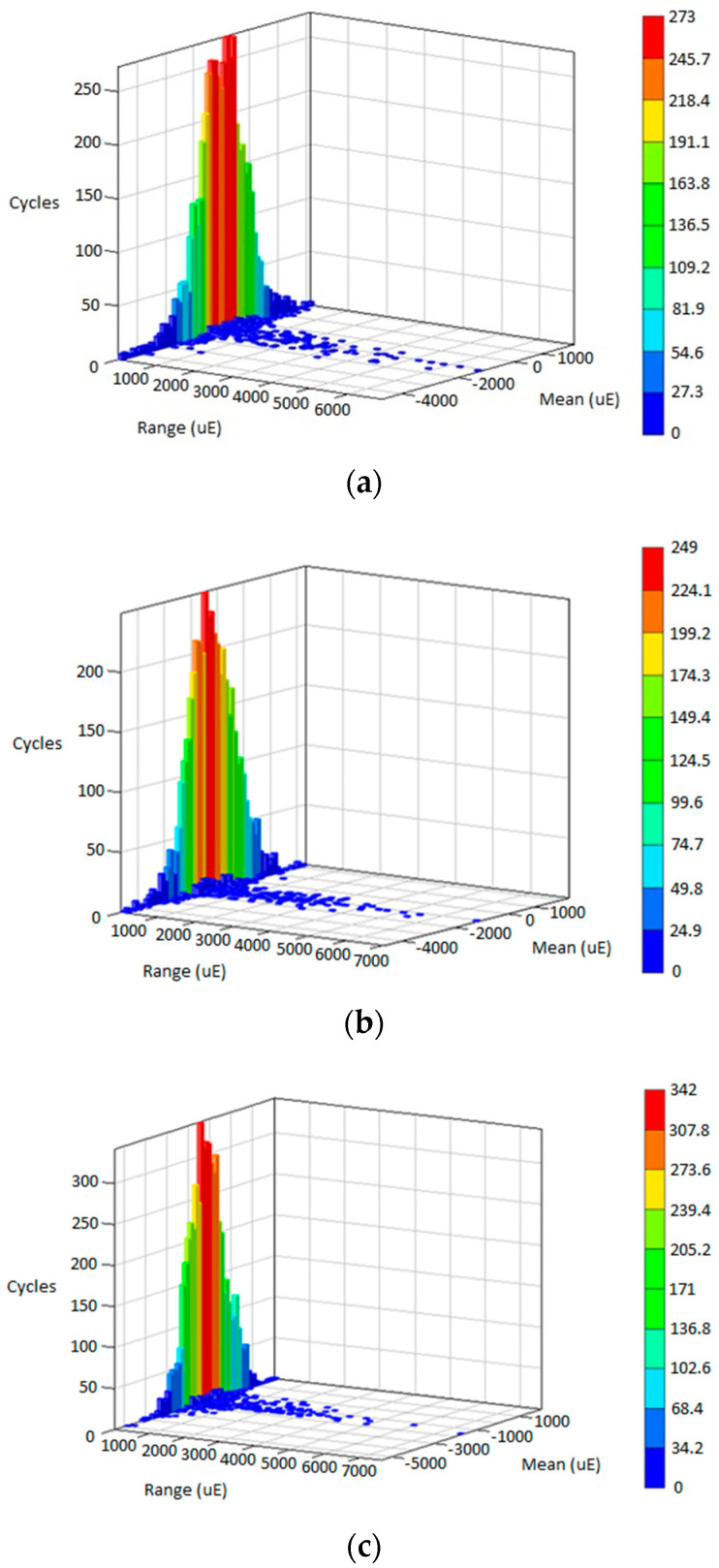
Rainflow cycle matrix for: (**a**) Highway D1; (**b**) Highway D2; (**c**) Highway D3.

**Figure 12:**
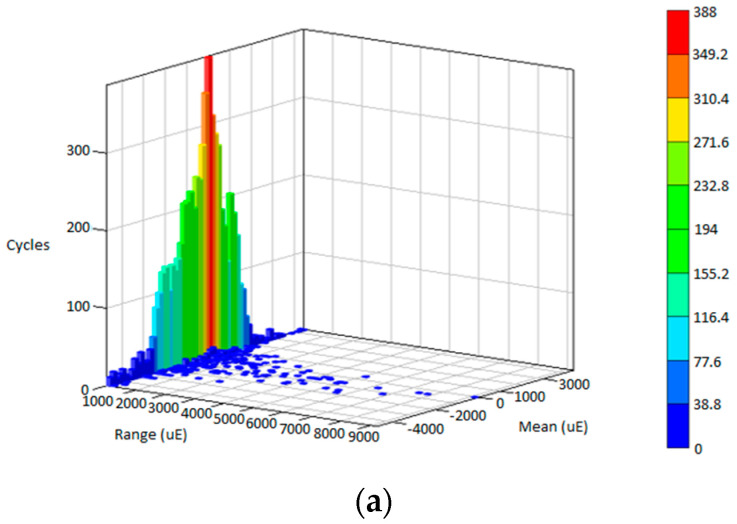

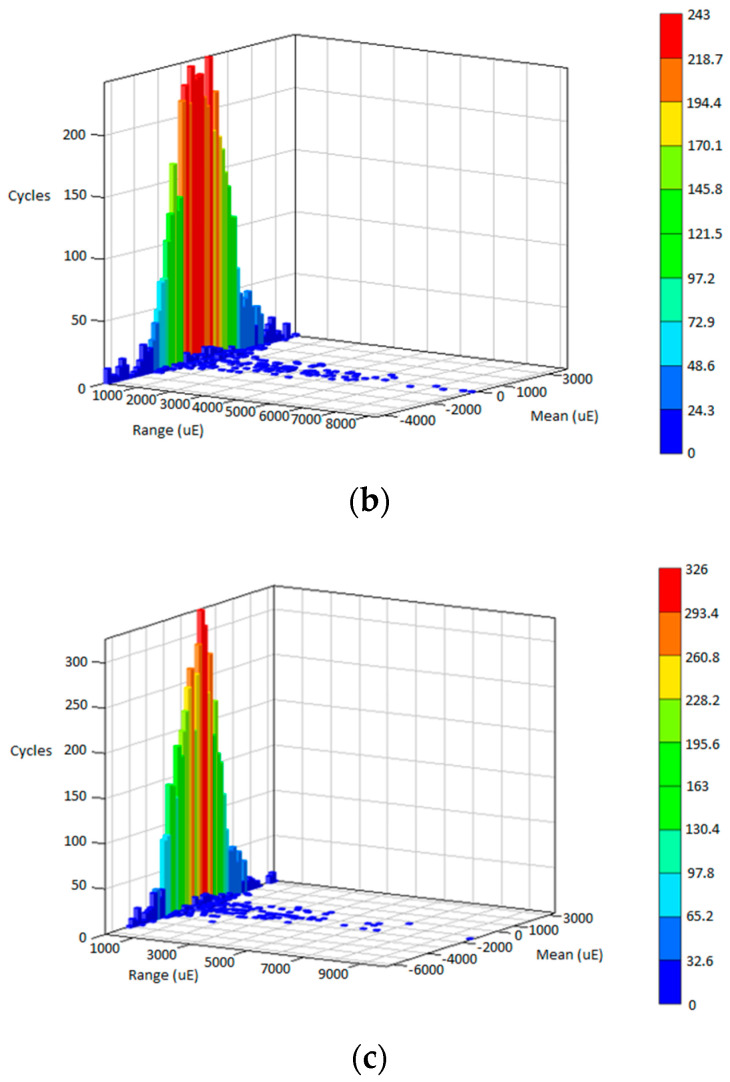
Rainflow cycle matrix for: (**a**) Rural D1; (**b**) Rural D2; (**c**) Rural D3.

**Figure 13:**
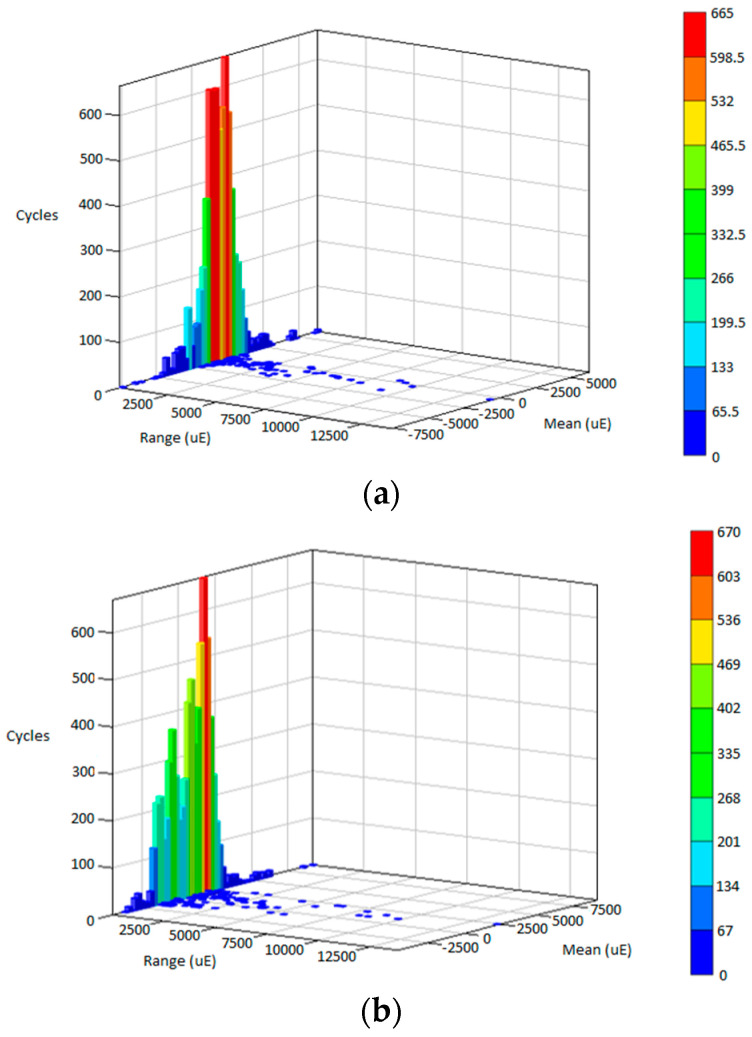

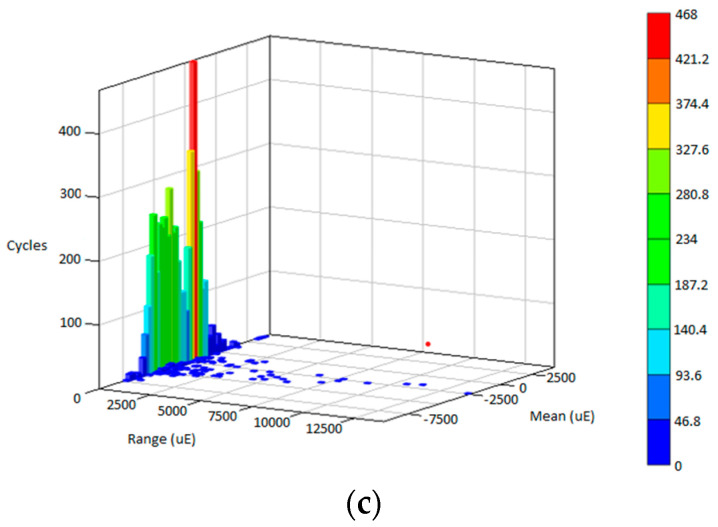
Rainflow cycle matrix for: (**a**) Campus D1; (**b**) Campus D2; (**c**) Campus D3.

The fatigue life is predicted in the time domain for every cycle by reversing the linear fatigue damage model in Equation (1) using the rainflow cycle count approach. Figure 11, Figure 12 and Figure 13 illustrate the rainflow cycle count for highway, rural and campus data, respectively. The histogram shows that highway data demonstrate the lowest maximum cycles compared with rural and highway data. The blue scatters in highway data show the minimum cycle at a lower range, whereas campus data exhibit the minimum cycle scattering at a higher range. This finding is related to random strain loads contained in the data influenced by the irregular road profiles. Hence, the strain load data of the leaf spring are scattered in the negative mean region where tension and compression conditions are described within the in-service life.

The fatigue life predicted using the strain–life models and ESD method is summarised in Table 2. The SWT model achieves the highest range in fatigue life compared with the Coffin–Manson, Morrow and ESD models. These values demonstrate that highway data obtained the maximum fatigue life due to its low amplitude range in strain data. Rural data indicated a slightly different trend in each strain–life approach, differing from campus data where the ESD model showed significant values compared with strain–life models. The fatigue life values indicated that the ESD model uses a different approach in assessing the cycle sequence effect compared with the conventional strain–life models that only consider mean stress effects. This finding can be related to the range in the rainflow cycle count where campus data obtained the highest range compared with highway and rural road data, as previously shown in Figure 12 and Figure 13.

**Figure 14:**
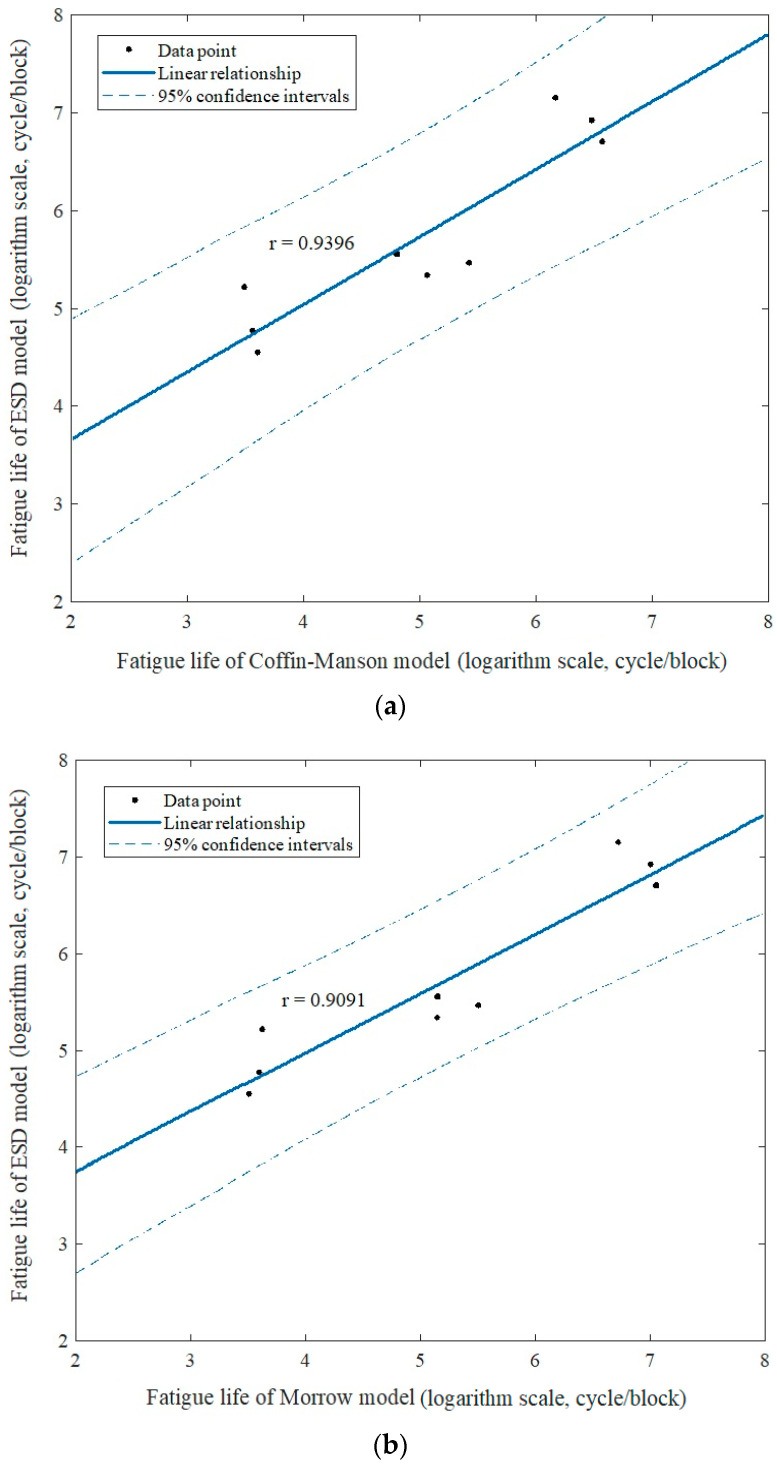

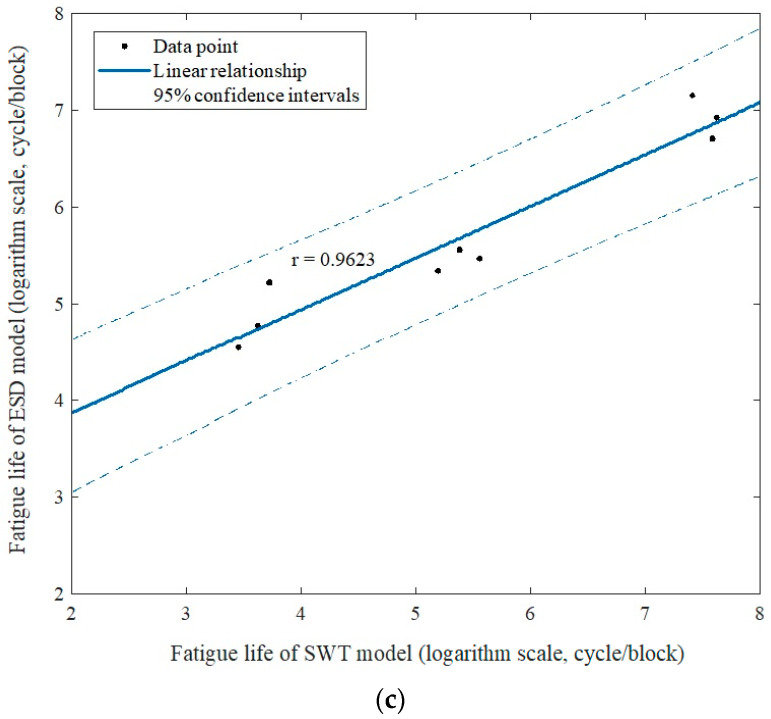
Pearson correlation for: (**a**) ESD and Coffin–Manson; (**b**) ESD and Morrow; (**c**) ESD and SWT.

In order to investigate the linearity of fatigue life data, the linear relationship of the Pearson correlation is used to correlate the ESD model with the established strain–life model. Figure 14 illustrates the Pearson correlation between the models of ESD versus Coffin–Manson, Morrow and SWT, respectively. The result shows the fatigue life data is within a 95% confidence interval. Additionally, the coefficient of Pearson correlation is calculated and obtained: the coefficient values, *r*, for Coffin–Manson, Morrow and SWT models are 0.9091, 0.9396 and 0.9623, respectively. These values indicate that the fatigue life data is significantly linear correlated, as the coefficient values exceed 0.9. A value greater than 0.8 is considered to represent good correlated data [43]. As a result, the ESD model is applicable to be used in predicting fatigue life.

**Figure 15:**
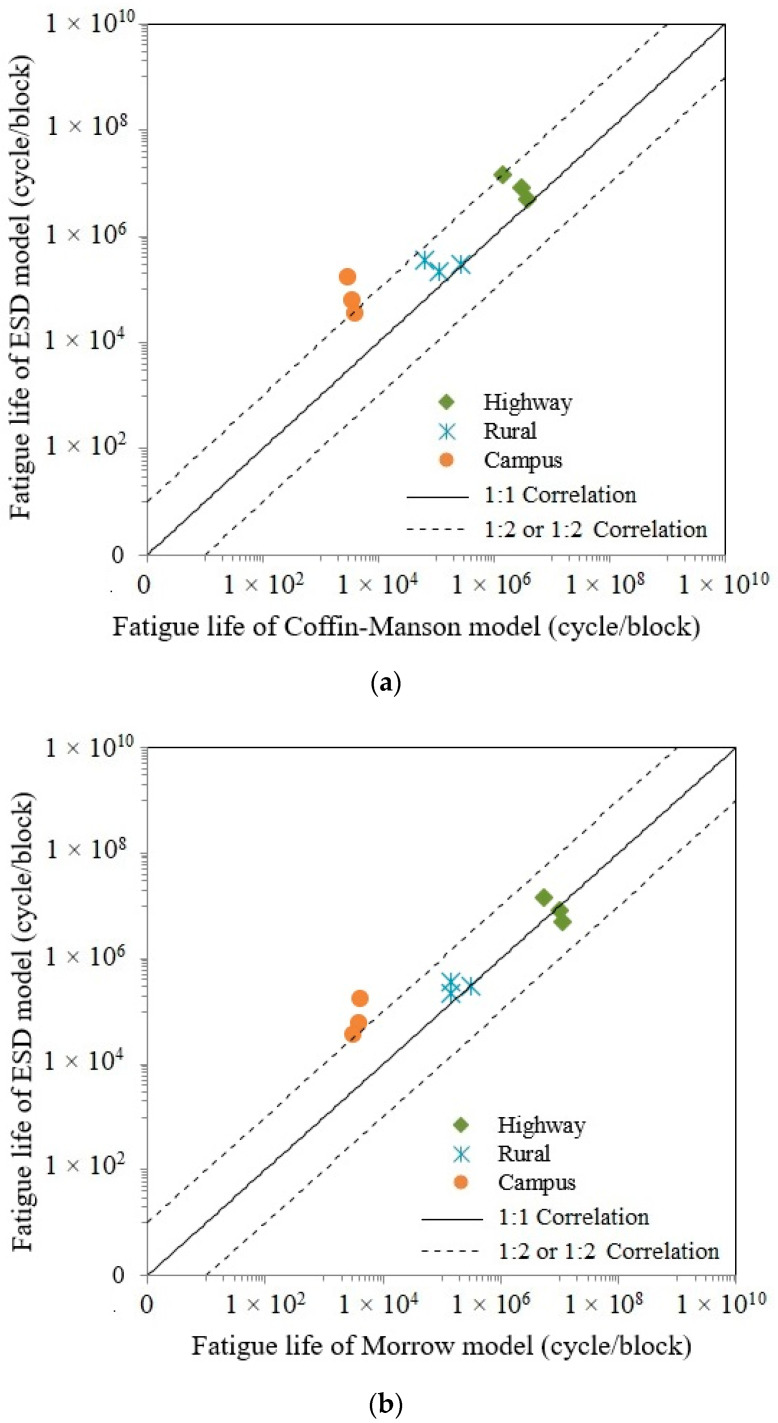

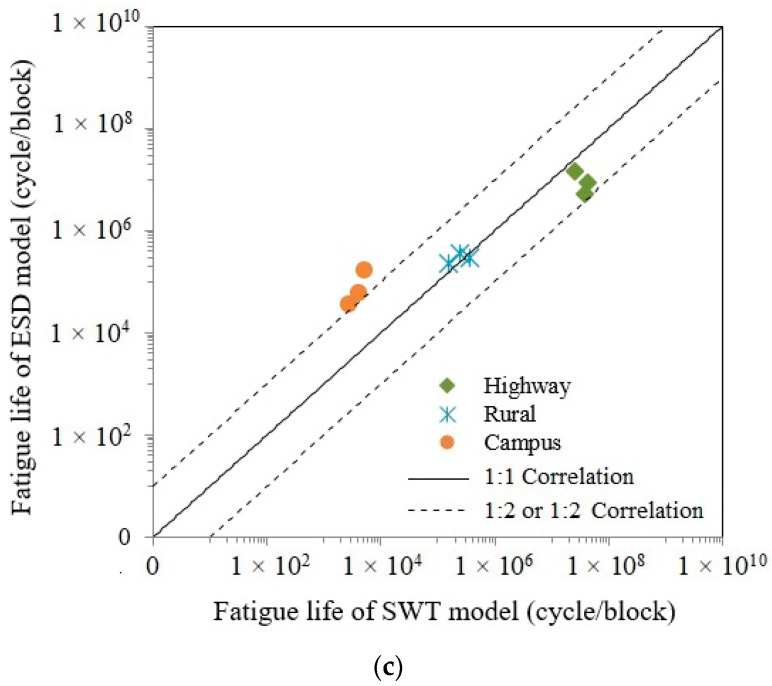
Fatigue life correlations for: (**a**) ESD versus Coffin–Manson; (**b**) ESD versus Morrow; (**c**) ESD versus SWT.

Figure 15 demonstrates the correlation based on a 1:2 or 2:1 fatigue correlation curve amongst the strain–life models of Coffin–Manson, Morrow and SWT with the ESD model. Campus data lying outside the 1:2 or 2:1 boundary line is likely due to several high amplitude ranges because of bumps and potholes. The prediction of fatigue life demonstrates a high correlation when fatigue life data lie within the 1:2 and 2:1 boundary line [43]. The ESD correlation between the Morrow and SWT models is superior to that of the Coffin–Manson model because several data lie outside the boundary lines. The Morrow and ESD correlation within fatigue life boundary conditions of 1:2 and 2:1 indicate the survivability of the predicted fatigue life values that can be used in fatigue reliability. The *R*^2^ value of Coffin–Manson versus ESD is 0.5661, while that of Morrow and SWT versus the ESD model are 0.6161 and 0.7181, respectively. The *R*^2^ values showed that the ESD model obtains the best correlation with the SWT model because it provides the highest *R*^2^ value versus those of the Morrow and Coffin–Manson models. Hence, the ESD model is proposed as it has nearly the same characteristics as SWT based on the presence of tension and compression during operating conditions.

### 3.4. Fatigue Reliability Assessment under Various Strain Loads

**Figure 16:**
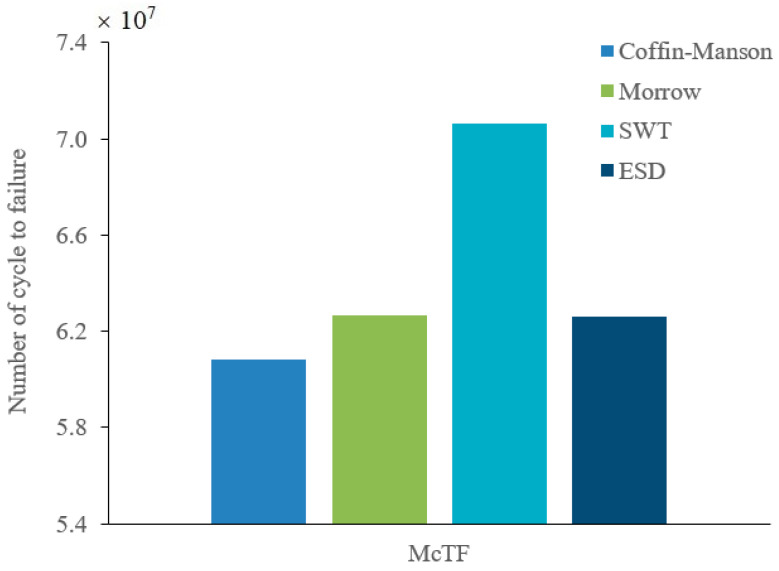
McTF for various strain–life models.

The Gumbel distribution was selected to assess the reliability of the leaf spring according to the normality test for fatigue strain data. Gumbel properties of location and scale parameters were calculated to estimate the mean value based on Equation (11). In reliability assessments, the mean time to failure (MTTF) is referred to as the mean value of Gumbel [21]. Because the fatigue life prediction is calculated subject to the cycles unit, the MTTF is therefore renamed as the mean cycle to failure (McTF) [59]. Figure 16 illustrates the McTF for various strain–life models, including the ESD model. The SWT model obtained the highest McTF value at 7.065 × 10^7^ cycle/block, followed by the Morrow and ESD models at 6.264 × 10^7^ and 6.259 × 10^7^ cycle/block, respectively, while the Coffin–Manson model demonstrated the lowest McTF value of 6.083 × 10^7^ cycle/block.

**Figure 17:**
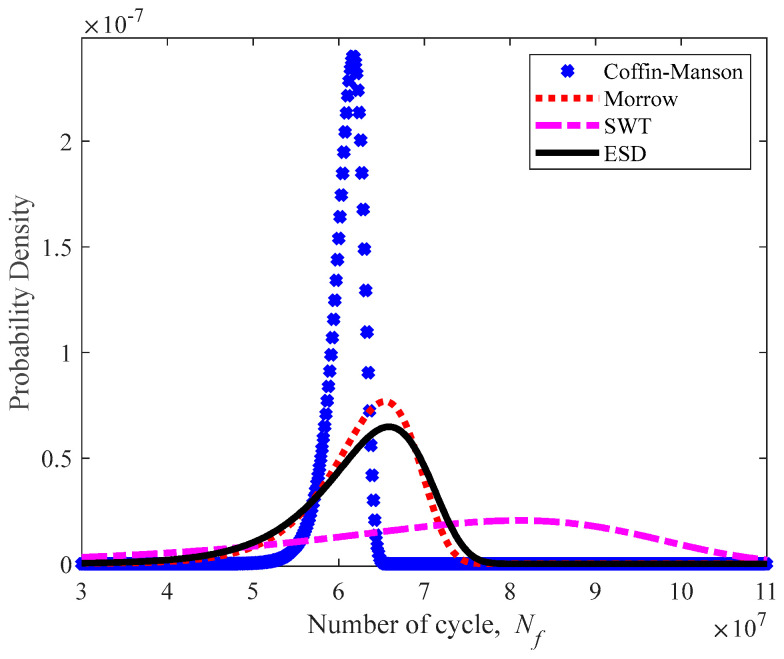
PDF plot for various strain–life models.

**Figure 18:**
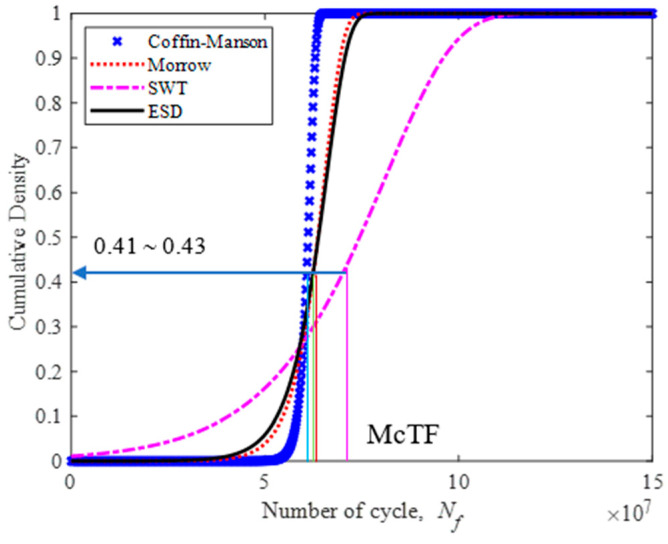
CDF plot for various strain–life models.

Figure 17 shows the PDF plots of durability models, indicating that the Morrow and ESD models are nearly identical. Likewise, the movement of the leaf spring case involves compressions and tensions during its in-service life. Therefore, amongst the strain–life models, Morrow and SWT are considered acceptable in value compared with ESD. This condition was asserted by Kadhim et al. [52], whereby the ESD model displayed a greater accuracy than the Coffin–Manson, Morrow and SWT models when incorporated into the estimation of fatigue life using numerous load data. The same behaviour was demonstrated by the CDF plot in Figure 18; the Morrow and ESD models describe a near-identical probability of failure within the range of 0.41 to 0.43.

**Figure 19:**
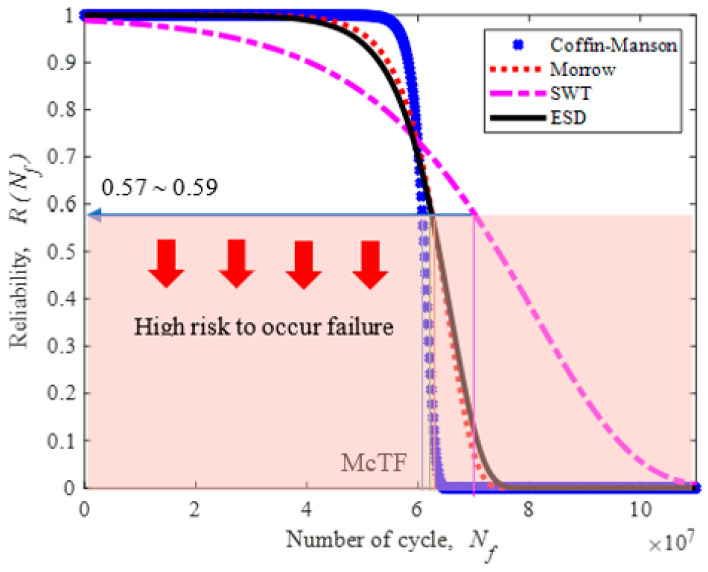
Reliability for various strain–life models.

**Figure 20:**
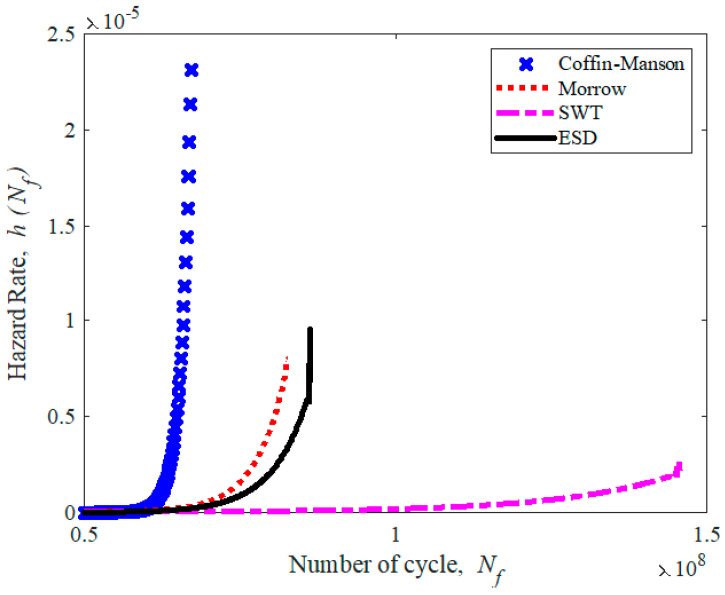
Hazard rate for various strain–life models.

The reliability of the fatigue lives data is within a range of 0.57 to 0.59. Therefore, the leaf spring would experience a high risk of failure when the reliability rate is below 0.59, as shown in Figure 19. The hazard rate of a leaf spring is illustrated in Figure 20. It shows a rapidly increasing hazard rate in the Coffin–Manson model until 6.72 × 10^7^ cycles/block at a rate of 2.3 × 10^−5^, followed by the increase in the Morrow and ESD models until 8.27 × 10^7^ and 9.5 × 10^−6^ cycles/block at a rate of 8.0 × 10^−6^ and 8.63 × 10^7^, respectively. The SWT model slowly increases at the maximum life of 1.46 × 10^8^ cycles/block at a rate of 2.8 × 10^−6^. Hence, this indicates that the leaf spring begins to deteriorate when the reliability value starts to fall below 0.59.

## 4. Conclusions

Fatigue life associated with the reliability assessment, based on a probabilistic method under various strain loads, and failure characterisation of the carbon steel heavy-vehicle leaf spring were discussed in this study. The fracture of the leaf spring was analysed via FESEM-EDX. The results showed that fatigue crack initiation starts at the U-bolt section. Similar to FEA, the deformation location occurred in the middle region of the leaf spring. The time history demonstrated that campus data typically contain the highest amplitude ranges compared with rural and highway data. This finding is consistent with the PDF plot and global statistical properties.

The highest fatigue life obtained from highway data was 4.19 × 10^7^ cycle/block via the SWT model, while that from rural and campus data was 4.81 × 10^5^ and 1.04 × 10^5^ cycle/block, respectively, based on the ESD method. Results indicated that strain–life models, including the ESD model, were within 1:2 and 2:1 conventional fatigue life, and they can be acceptably used in predicting fatigue life. The reliability assessment based on the Gumbel distribution demonstrated that the exposed leaf spring might experience a high risk of failure when the reliability rate exceeds 0.59 and a low rate of hazard at a range of 9.6 × 10^−8^ to 1.2 × 10^−7^ when the McTF point is reached. Hence, the ESD model can successfully estimate the fatigue life in assessing leaf spring reliability under various road load conditions.

## Figures and Tables

**Table 1:** Chemical composition of SAE 5160 material (wt %).

c	Mn	S	Cr	V
0.52	0.5	0.3	0.2	0.1

**Table 2:** Fatigue life prediction of various road conditions.

Road	Coffin–Manson (Cycle/Block)	Morrow (Cycle/Block)	SWT (Cycle/Block)	ESD (Cycle/Block)
Highway D1	3.02 × 10^6^	1.01 × 10^7^	4.19 × 10^7^	1.75 × 10^6^
Highway D2	3.73 × 10^6^	1.13 × 10^7^	3.84 × 10^7^	2.15 × 10^6^
Highway D3	1.46 × 10^6^	5.26 × 10^6^	2.57 × 10^7^	2.61 × 10^6^
Rural D1	1.15 × 10^5^	1.40 × 10^5^	1.56 × 10^5^	3.44 × 10^5^
Rural D2	2.65 × 10^5^	3.19 × 10^5^	3.60 × 10^5^	3.11 × 10^5^
Rural D3	6.37 × 10^4^	1.41 × 10^5^	2.40 × 10^5^	4.81 × 10^5^
Campus D1	3.63 × 10^3^	3.96 × 10^3^	4.19 × 10^3^	1.04 × 10^5^
Campus D2	4.03 × 10^3^	3.21 × 10^3^	2.85 × 10^3^	6.96 × 10^4^
Campus D3	3.08 × 10^3^	4.21 × 10^3^	5.28 × 10^3^	9.85 × 10^4^
